# Early diagnosis of jaw osteomyelitis by easy digitalized panoramic analysis

**DOI:** 10.1186/s40902-019-0188-2

**Published:** 2019-02-01

**Authors:** Moo Soung Park, Mi Young Eo, Hoon Myoung, Soung Min Kim, Jong Ho Lee

**Affiliations:** 1 Department of Dentistry, School of Dentistry, Dental Research Institute, Seoul National University, Seoul, South Korea; 2 Department of Oral and Maxillofacial Surgery, Dental Research Institute, School of Dentistry, Seoul National University, 101, Daehak-ro, Jongno-gu, Seoul, 110-768 South Korea

**Keywords:** Early diagnosis, Digitalized panoramic analysis, Osteomyelitis of jaw, Decision making tree, Picture archiving and communication system (PACS)

## Abstract

**Background:**

Osteomyelitis is an intraosseous inflammatory disease characterized by progressive inflammatory osteoclasia and ossification. The use of quantitative analysis to assist interpretation of osteomyelitis is increasingly being considered. The objective of this study was to perform early diagnosis of osteomyelitis on digital panoramic radiographs using basic functions provided by picture archiving and communication system (PACS), a program used to show radiographic images.

**Methods:**

This study targeted a total of 95 patients whose symptoms were confirmed as osteomyelitis under clinical, radiologic, pathological diagnosis over 11 years from 2008 to 2017. Five categorized patients were osteoradionecrosis, bisphosphonate-related osteonecrosis of jaw (BRONJ, suppurative and sclerosing type), and bacterial osteomyelitis (suppurative and sclerosing type), and the control group was 117 randomly sampled. The photographic density in a certain area of the digital panoramic radiograph was determined and compared using the “measure area rectangle,” one of the basic PACS functions in INFINITT PACS® (INFINITT Healthcare, Seoul, South Korea). A conditional inference tree, one type of decision making tree, was generated with the program R for statistical analysis with SPSS®.

**Results:**

In the conditional inference tree generated from the obtained data, cases where the difference in average value exceeded 54.49 and the difference in minimum value was less than 54.49 and greater than 12.81 and the difference in minimum value exceeded 39 were considered suspicious of osteomyelitis. From these results, the disease could be correctly classified with a probability of 88.1%. There was no difference in photographic density value of BRONJ and bacterial osteomyelitis; therefore, it was not possible to classify BRONJ and bacterial osteomyelitis by quantitative analysis of panoramic radiographs based on existing research.

**Conclusions:**

This study demonstrates that it is feasible to measure photographic density using a basic function in PACS and apply the data to assist in the diagnosis of osteomyelitis.

**Electronic supplementary material:**

The online version of this article (10.1186/s40902-019-0188-2) contains supplementary material, which is available to authorized users.

## Background

Osteomyelitis is an intraosseous inflammatory process including the cortex bone and periosteum that is characterized by progressive inflammatory osteoclasia with ossification [1–3]. Osteomyelitis can occur in humans at any site of the bone including the femur, humerus, or jaw. The most typical pathogenesis is infection with bacteria such as *Staphylococcus aureus* or *Mycobacteria*, but it may also be induced by trauma, radiation, or specific drugs [4, 5].

Typical clinical symptoms of patients with osteomyelitis are edema and/or pain and formation of fistula. Radiologically, the condition demonstrates a radiopaque image with an unclear boundary [6]. Early administration of antibiotics with an accurate diagnosis is the best approach to treatment of osteomyelitis, and intravenous injection rather than oral medication sometimes shows better prognosis. If osteomyelitis is confirmed by tissue biopsy, a surgical approach of removing the infection source and pus and concurrent administration of antibiotics is recommended for improved prognosis. However, if early diagnosis is not achieved or suitable antibiotics are not used, the prognosis may prove to be unfavorable due to the increase of focus or spread of infection to other bone sites [7].

Osteomyelitis of the jaw is an important disease accounting for a considerable proportion of patients visiting oral and maxillofacial surgery departments despite technical advancements in the dental field and the development of antibiotics [7]. Development of medical science and increased oral sanitation has contributed to reducing the prevalence of jaw osteomyelitis over the past couple of decades. Recently, a new type of maxillary osteomyelitis called bisphosphonate-related osteonecrosis of the jaw (BRONJ) or osteoradionecrosis (ORN), which is induced primarily by the drug bisphosphonate and secondarily by radiotherapy, has been described [8]. BRONJ was first reported by Marx in 2003, and since 2006, its disease etiology and prognosis have been extensively reported [8, 9].

At present, diagnosis of osteomyelitis is primarily performed through panoramic radiography, oral cavity photography, and clinical diagnostic examination [7]. Among these, this study pays particular attention to the role of panoramic radiography. Since its first development in 1991, digital panoramic radiography has been effectively used for general examination to confirm the structure and condition of the maxillary bone [10]. Among traditional plain radiographs, panoramic radiograph is the sole means of obtaining information on the maxilla and mandible at the same time. For this reason, it is used for identifying and diagnosing general conditions of the maxillary bone and plays a pivotal role in diagnosing osteomyelitis even though it is unable to diagnose the condition independently [11, 12]. Findings of osteomyelitis that can be observed in panoramic radiograph include increased thickness of alveolar lamina dura, sclerogenic variation around mandibular canal, sclerogenic variation of the maxillary bone, and confirmation of osteoclasia and bone pattern [7]. These characteristics are findings that can be confirmed in general osteomyelitis, but at an early stage of 4–8 days after onset of osteomyelitis such signs may not be found in diagnostic radiographs [13]. The hospital staff decide whether to perform additional radiographic examination or clinical examination based on the reports of relevant medical staff.

This study aimed to support the role of dentists by providing an osteomyelitis diagnosis key through a quantitative approach performed in a relatively simple manner. In particular, the objective of this study was to allow early diagnosis of osteomyelitis on digital panoramic radiographs using basic functions provided by the picture archiving communication system (PACS) program showing radiographic images.

## Methods

This study targeted a total of 98 patients whose symptoms were confirmed as osteomyelitis under clinical, radiologic, pathological diagnosis at the Department of Oral and Maxillofacial Surgery in the Seoul National University Dental Hospital (SNUDH) over 11 years from 2008 to 2017. Cases that were clinically diagnosed as osteomyelitis but were not radiologically diagnosed due to technical limitations were not included in the study. After exclusion of three cases that were concluded to be non-osteomyelitis under clinical and pathological diagnosis even though the possibility of osteomyelitis was suggested radiologically, a total of 95 patients were analyzed in the final research.

Patients with osteomyelitis were classified under clinical, radiological, and pathological diagnosis into five categories: ORN as group A; BRONJ, suppurative type as group B1 or sclerosing type as group B2; bacterial infectious osteomyelitis, suppurative type as group C1 or sclerosing type as group C2. For subclassification of BRONJ, cases where fistula or pus were formed and necrotized osseous tissue was clearly exposed were classified as suppurative and cases where sclerosing osseous tissue was exposed without necrotized tissue present were classified as sclerosing type. The collection and processing of this clinical data was approved by the institutional review board (IRB S-D20160039) of Seoul National University.

### Data acquisitions

**Fig. 1:**
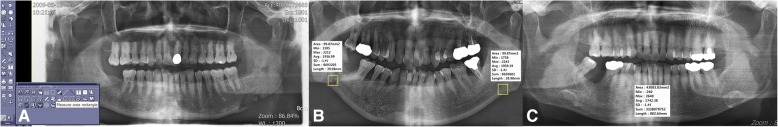
Measurement method using “measure area rectangle” of INFINITT PACS® (INFINITT Healthcare, Seoul, South Korea) of bacterial osteomyelitis patient number 10 (**a**), comparison of photographic density in a patient with osteomyelitis for a focus in left mandible and control in right mandible of bacterial osteomyelitis patient number 12 (**b**), and maximum and minimum photographic density in panoramic radiograph of bacterial osteomyelitis patient number 17 (**c**)

Photographic density of a selected area in the digital panoramic radiograph was determined by using the “measure area rectangle” function, one of the basic functions in INFINITT PACS® (INFINITT Healthcare, Seoul, South Korea) used at *** (Fig. 1a). When using the measure area rectangle function, minimum, maximum, and average values of photographic density in a certain area could be measured from panoramic radiograph and computed tomography (CT) images. For a certain designated area, the area, min, max, avg., standard deviation (SD), sum, and length values can be deduced.

**Fig. 2:**
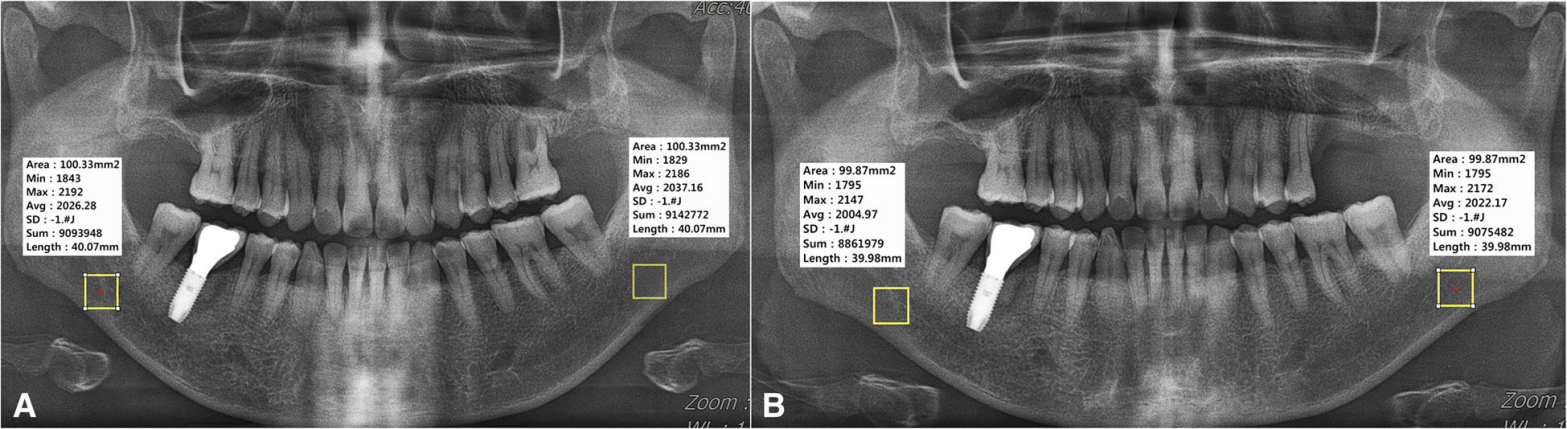
Comparison of photographic density in normal control group number 9 (**a**), and panoramic radiograph of the same patient taken another day (**b**). These figures were used for only method description from unrelated research data in osteomyelitis patients

Independent variables that could be directly designated by the user include area and length, and if these factors are controlled, a region of interest (ROI) of the same size for each digital panoramic radiograph could be designated (Fig. 1b). Min, max, and avg. are deduced as dependent variables and expressed as values between − 240 and 2640; low and high photographic density values represent radiopaque and radiolucent images, respectively (Fig. 1c). We used the center of lesion. If there is a sequestrum in the suppurative type of osteomyelitis, we selected this suppurative part as the center of lesion, because it was difficult to see this sequestrum as a center of lesion. SD is the same value if the additional option is not requested separately, and sum means the total photographic density value of all the pixels in the area. These two values were not used in this study (Fig. 2a).

In the process of deducing photographic density, its value is represented as Hounsfield units in CT and as raw values in panoramic radiograph. In the case of CT, the photographic density is readjusted as Hounsfield units through a process of standardization every day such that water has a value of zero. Therefore, it could be assigned by absolute value only. However, in radiography, there is no such standardization process and comparison of absolute values among different images is meaningless due to error in the process of moving osseous tissue from 3D to 2D depending on posture or the angle at the time of image photographing. In particular, the same value is not represented when photographing the same patient twice, even when photographic density at the same position is measured (Fig. 2b). On the other hand, comparing each part in one sheet of a single panoramic photograph is considered relatively standardized. Comparing photographic density of two different parts in one image should be sufficiently standardized that clinicians can use panoramic radiographs in the diagnostic process.

Under this background, in this study, photographic density of both sides was compared by dividing the digital panoramic radiograph of patients with osteomyelitis based on the median line. Min, max, and avg. values in each area were recorded by designating the same rectangular ROI of 100 mm^2^ to opposite maxillary bones based on the median line after first designating the ROI based on the focus. Any error due to manual designation of the ROI was minimized by generating one panoramic radiograph with a rectangular ROI with length and width designed to give an area of 100 mm^2^ with rounding to the first digit of decimal point. Because the value obtained in this manner is not meaningful as an absolute value, the difference values obtained by subtracting the min, max, and avg. values of the normal area from those of the focus area were designated as the representative values of each image.

To compare each image result for the final obtained difference value, we also compared left/right sides in normal digital panoramic radiographs of individuals without osteomyelitis as the normal control group. This group included 117 randomly sampled people who visited **** from 2008 to 2017 and did not receive a diagnosis of osteomyelitis. The control group consisted 59 males and 58 females with average age of 48.9 years. Among patients sampled as the control group, 45% visited for implant, 40% for tooth extraction, and the remaining 15% for maxillary sinus surgery, plate removal, or curettage. In the control group, photographic density was measured at symmetric points by dividing the panoramic radiograph into left, and right in the same way as for the patients with osteomyelitis. Differences in left and right side min, max, and avg. values obtained by measurement were designated as representative values of each image.

### Statistical processing

All obtained data were statistically processed using a decision making tree and *t* test. The decision making tree is one of the data mining analysis techniques and is a method of predicting and classifying the target group to be researched into small groups based on decision making rules. As the analysis process is classified and expressed by a tree structure, it has the advantage that the analysis method can be easily understood and explained compared with analysis methods of discriminant analysis, regression analysis, or neural networks [14].

The decision making tree is branched to the left side in case of answering “Yes” to the question of “Is variable X smaller than constant c when comparing the former with the latter?” and to the right side in case of “No.” Each branched unit is called a node; the relative superordinate node is called a parent node and the subordinate node is a child node. Branching should always be achieved so that the purity of the child node is higher than that of the parent node [15]. We tried to simplify verification of the significance of a newly generated decision making tree using prepared data followed by exclusion of certain parts of the total data. Error was briefly solved by setting data quantity as 0.8, that is 80% in the interactive mode analysis of R program®, and by using the remaining 20% data, type 1 and 2 errors were verified through future significance verification.

In this study, a conditional inference tree was generated using R program® version 3.2.3. To compare the average of two samples extracted from a different population, *t* test was performed using SPSS® version 21 (release 21.0.0.0). The *t* test is a method of verifying whether two groups show a statistically significant difference and can utilized when the variance of the population is unknown. Significance probability was set as 0.01 (*p* < 0.01), and in all the comparisons, the null hypothesis was defined as no difference among mean values extracted from each group.

## Results

Clinicopathologic data of patients with osteomyelitis with normal control group are presented in Additional file 1: Table S3–S6. Among the total 95 patients who visited for osteomyelitis, 35 were males, 60 were females, and the average age was 63.52 ± 15.93 years; this was significantly different from the randomly selected normal patient group (Table 1). Thus, it is certainly randomizing when selecting patients in this study. The most common affected site of osteomyelitis was right mandible (46.3%, *n* = 44), followed by left mandible (31.6%, *n* = 30). When dividing mandible/maxilla into two arches, disease occurred more often in the mandible (86.3%, *n* = 82) than maxilla (12.7%, *n* = 12) and was present in both mandible and maxilla for one patient (Additional file 1: Table S7). For patients with bacterial osteomyelitis only, the average age was 58.42 ± 16.45 and the most commonly affected site was right mandible (46.7%, *n* = 28). In cases of BRONJ, the average age was 73.19 ± 9.03 and the most affected site was the right mandible (45.2%, *n* = 14). In cases of osteoradionecrosis, the average age of 65 ± 14.71 and the most affected site was right mandible (50%, *n* = 2). The most frequently performed therapeutic method was saucerization (*n* = 52), and medication control was followed by mandibulectomy and maxillectomy (*n* = 7) (Table 2).

**Table 1:** Statistical significance verification of age distribution between osteomyelitis patient and control group

Independent Samples Test										
		Levene's Test for Equality of Variances		t-test for Equality of Means						
		F	Sig.	t	df	Sig. (2-tailed)	Mean Difference	Std. Error Difference	95% Confidence Interval of the Difference	
									Lower	Upper
avg	Equal variances assumed	3.438	0.065	-6.087	210.000	0.000	-14.618	2.401	-19.352	-9.884
	Equal variances not assumed			-6.176	209.011	0.000	-14.618	2.367	19.284	-9.953

p < 0.01, reject null hypothesis and there is difference between two groups

**Table 2:** Treatment of osteomyelitis patient

Treatment	Osteoradio-necrosis	BRONJ	Bacterial osteomyelitis	Total (osteomyelitis)
Saucerization	3	21	26	50
Medicine	0	2	6	8
Mandibulectomy / maxillectomy	1	2	4	7
Sequestrectomy	0	3	3	6
Mass resection	0	1	1	2
Incision and Drainage	0	1	2	3
Extraction	0	0	3	3
Cyst enucleation	0	0	3	3
Endodontic treatment	0	0	1	1
Untreated	0	1	11	12
Total	4	31	60	95

### Comparison between osteomyelitis patient group with control group

**Fig. 3:**
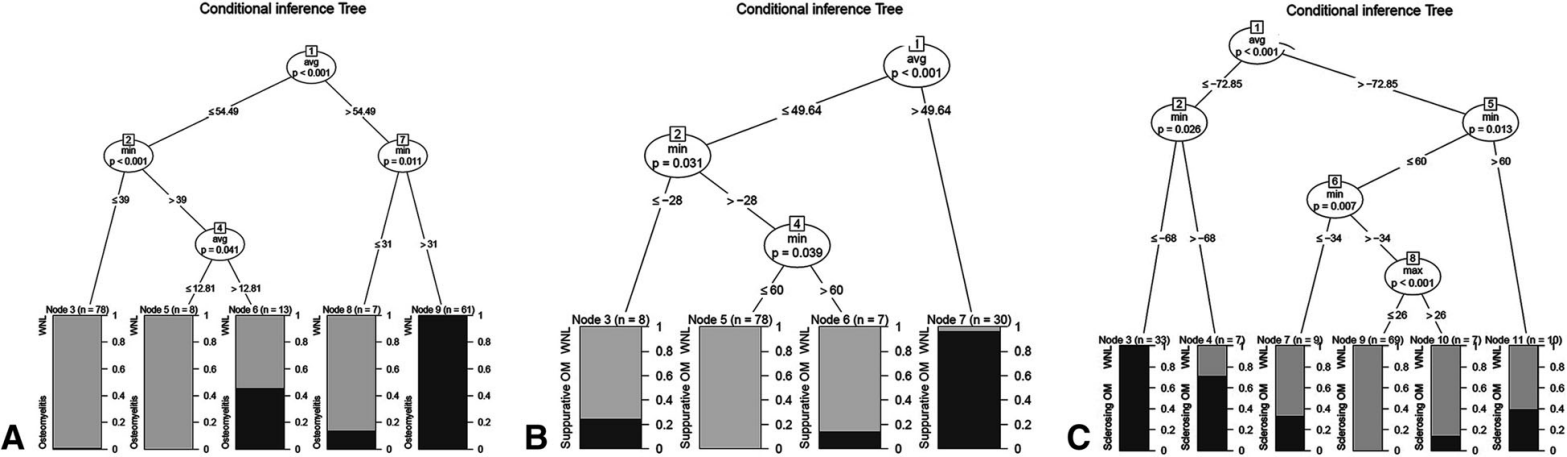
Conditional inference trees showing comparison between osteomyelitis patient and control group (**a**), between suppurative bacterial osteomyelitis, suppurative BRONJ, and control group (**b**), between osteoradionecrosis, sclerosing bacterial osteomyelitis, sclerosing BRONJ, and control group (**c**)

Data for all 95 osteomyelitis patients under three hierarchical classifications were compared with data for 114 controls (*n* = 209). After exclusion of data for 42 patients that were randomly sampled for significance test, data for a total of 167 patients were used for formation of the decision making tree. All the raw materials were analyzed by taking absolute values (Fig. 3a).

First branching of the conditional inference tree was achieved through confirmation of whether the difference in average value of photographic density exceeded 54.49. In cases exceeding this value, second branching was achieved through confirmation of whether the difference in minimum value of photographic density exceeded 31. Among 62 cases exceeding 31, 100% were confirmed as osteomyelitis. In cases with the difference in min value below 31, 85.7% (*n* = 6) were proved to be normal and 14.3% (*n* = 1) had osteomyelitis. At the first branching, when the difference in average value was below 54.49, second branching was achieved through confirmation whether the difference in min value exceeds 39. Among cases with difference in min value below 39, 98.7% (*n* = 77) were normal and 1.3% (*n* = 1) were osteomyelitis. In cases in which the difference in min value was below 39, final branching was performed by confirming whether the average value difference exceeds 12.81. In a node where the difference in average value was below 12.81, 100% (*n* = 8) were normal whereas among cases where the difference in average value difference was between 12.81 and 54.49, 53.8% (*n* = 7) were normal and 46.2% (*n* = 6) were represented as osteomyelitis.

At the time of verifying significance, 37 among a total of 42 data were classified without error and five cases of osteomyelitis were mistakenly classified as normal (Additional file 1: Table S8). This result means that when using the above conditional inference tree, correct classification could be performed with a probability of 88.1%.

### Comparison between B1, C1, and control

In bacterial osteomyelitis and BRONJ groups, 40 data applicable to suppurative type osteomyelitis were compared with 114 normal control. A decision making tree was generated using a total of 123 data, excluding 20% for significance verification (Fig. 3b).

The conditional inference tree was first classified as suppurative type osteomyelitis (C1) if the difference in average value of photographic density exceeded 49.64. In the node branched like this, osteomyelitis was represented at a rate of 96.7% (*n* = 29) and normal at 3.3% (*n* = 1). The group with difference in average value ≤ 49.64 was branched into two nodes depending on whether the difference in min value was greater than − 28. The node with difference in min value less than − 28 contained 75% control (*n* = 6) and 25% C1 (*n* = 2).

In cases where the difference in minimum value exceeded − 28, one more branching was represented depending on whether the difference in min value was greater than 60. In the node in which the difference in min value was greater than 60, 85.7% (*n* = 6) were classified as control and 14.3% (*n* = 1) as C1. Finally, the node in which the difference in min value was between − 28 and 60 consisted of only control cases (100%, *n* = 78).

At the time of significance verification, 28 data were classified without error among a total of 31 data; in the remaining three cases, C1 was mistakenly classified as control. This result means that when using conditional inference tree, correct classification could be performed based on a probability of 96.8%.

### Comparison between A, B2, C2, and control

For two groups including ORN/bacterial osteomyelitis and BRONJ, 55 data applicable to sclerosing type osteomyelitis were compared with 114 normal patient data as the control group (Fig. 3c).

The conditional inference tree classified the first node depending on whether the difference in avg. value of photographic density exceeded − 72.85. Cases where the difference in avg. value was below − 72.85 were branched to the final node depending on whether the difference in min value exceeded − 68. Among cases with the difference in avg. value below − 72.85 and difference in min value below − 68, 100% (*n* = 33) were classified as C2. If the difference in min value exceeded − 68, 28.6% (*n* = 2) were normal and 71.4% (*n* = 5) were C2.

When the difference in avg. value exceeded − 72.85, the first node was branched again by confirming whether the difference in min value exceeded 60. In cases where the difference in avg. value exceeded − 72.85 and the difference in min value exceeded 60, 60.0% (*n* = 6) were classified as normal and 40% (*n* = 4) as C2. Cases with difference in min value below 60 were additionally branched by confirming whether the difference in min value exceeded 34.

In case where the difference in min value was below − 34, 66.7% (*n* = 6) were classified as control and 33.3% (*n* = 3) as C2. Cases where the difference in min value was over − 34 and below 60 were branched once more by confirming whether the difference in min value finally exceeds 26. In cases with difference in max value below 26, 100% (*n* = 69) were normal and in cases with difference in max value exceeding 26, 85.7% (*n* = 6) were classified as normal and 14.3% (*n* = 1) as C2.

At the time of significance verification, 32 among 34 data were classified without an error and two normal cases were mistakenly classified as C2. This result means that when using above conditional inference tree, correct classification could be performed based on a probability of 94.1%.

### Statistical significance verification between B1 + C1 and B2 + C2

*T* test was performed based on a null hypothesis that there is no difference in avg., max, and min values in two groups of suppurative type B1 + C1 and sclerosing type B2 + C2 (Additional file 1: Table S9–S11). As the significance probability for avg., max, and min values was > 0.01 for the two groups, the above null hypothesis was dismissed. There is therefore a respective difference in the avg., max, and min values in the above two groups.

### Statistical significance verification among B1 and C1

*T* test was performed based on a null hypothesis that there are no differences in the avg., max, and min values of the two groups of BRONJ group showing suppurative type OM and bacterial osteomyelitis group showing suppurative type (Additional file 1: Table S12–S14).

As the significance probability of the avg., max, and min values of the two groups was < 0.01, the above null hypothesis is dismissed. There is no difference in avg., max, and min values of the above two groups.

### Statistical significance verification among B2 and C2

*T* test was performed based on a null hypothesis that there are no differences in the avg., max, and min values of the two groups of B2 and C2 (Additional file 1: Tables S15–S17). As the significance probability of the avg., max, and min values of these two groups was < 0.01, the above null hypothesis is dismissed. There are no differences in the avg., max, and min values of the above two groups.

## Discussion

Osteomyelitis of the jaw was most common in females (average age 63.52 ± 15.93; 63.2%, *n* = 60) and in the in mandible (86.3%, *n* = 82), which is consistent with the fact that the morbidity of osteomyelitis in the maxilla is lower than that in the mandible because blood circulation is relatively rich in the maxilla [16].

### Interpretation of clinical and radiographic analyses of osteomyelitis

Before a method of providing tomographic images such as CT was available, panoramic radiograph was the sole means of diagnosis and prognosis follow-up. By high-definition CT, the resection level of necrotized bone can be determined by identifying the necrosis level of the cortex bone or position of sequestration. However, panoramic radiography remains an excellent supplementary approach that delivers a lot of information at an early stage and has an excellent role in observing prognosis.^7^ The development of PACS has further strengthened prognosis observation. PACS is a computer-based system designed to make the diagnostic process easy. Diagnosis is supported by ensuring ready access to data by attaching Digital Imaging and Communications in Medicine (DICOM) including X-ray, MRI, or CT to the electronic medical record (EMR) of the patients and having the operator confirm the contents read by the reader [17]. Research results show improved efficiency in the diagnostic process since the introduction of PACS [18]. Marketing of this system was first developed in 1980 and has been grown consistently; at present, many university hospitals have adopted this system and its use in dental clinics is increasing [17, 19]. If findings of osteomyelitis are found in patients visiting dental clinic as a result of diagnosis through panoramic radiograph and ocular inspection, referral to a hospital is one of the important duties of the dentist. However, as CT equipment or a separate image dentistry reader for detailed examination is rarely available in dental clinics, there may be a difficulty in detecting osteomyelitis. In addition, when considering osteomyelitis using an image dentistry reader at a dental clinic, consideration of evidence supporting such readings would increase the reading accuracy.

A decision making tree was generated by comparing a patient group with osteomyelitis with a normal control group using differences in the average and minimum values of radiographic density as the classification standard (Fig. 3a). In the conditional inference tree generated using obtained data, cases where the difference in average value exceeded 54.49 and the difference in min value was between 54.49 and 12.81 and min value difference exceeded 39 were suspicious of osteomyelitis. The fact that disease could be correctly classified with a probability of 88.1% as a result of significance verification using each conditional inference tree suggested in Fig. 3a means that this approach does not significantly deviate from the existing common idea of preparing a report with a probability of 90% at the time of reading radiography. In cases where a function that may assist the accuracy of reading radiography data cannot be performed or a reading expert is unable to perform reading, its substituted mean is considered to be available.

When reading existing radiographic images of osteomyelitis qualitatively, attention shall be paid to the following characteristics. Imaging findings that are clearly different from normal in panoramic images are characteristics of both acute and chronic disease, and the respective pathologic patterns are different. In acute osteomyelitis, loss of spongy trabecular bone structure on local radiographic images is represented first. This change in spongy bone pattern is one of the key elements when reading osteomyelitis qualitatively [20]. This change may be accompanied by an extension of periodontal ligament space or loss of alveolar lamina dura in the tooth. Radiographic findings in which the boundary is not clear or is obscured are sometimes observed. In this case, osteomyelitis is considered to have passed to the chronic stage [7, 21]. This reading result depends on the experience of experts and as this qualitative reading is not perfect, it may need to be supplemented.

In comparisons of other reading results, there is no report on the reading error of osteomyelitis, but only 50% consensus was achieved when dental root apex-related radiographic images of 253 cases were read by two conservative dentistry specialists, three 2nd term residents of conservative dentistry, and a radiology professor. In addition, when evaluating these cases after 6–8 months, only 75–83% of the readers made the same diagnosis as the first reading [22]. When 24 sheets of panoramic images were given to a total of 12 persons including three each of oral surgery specialist, pathology specialist, radiology specialist, and dental doctor and the readers were asked to choose which one among four kinds of solitary focus (ameloblastoma, keratocyst, dentigerous, and traumatic bone cyst) was applicable to each image, only 56% read the image correctly regardless of additional post-treatment by computer [23].

Existing research on radiographic reading of osteomyelitis is limited. According to one research study of reading acute osteomyelitis radiographically, during the first 2 weeks, three out of four sheets of radiographic photographs showed normal images, and even when extended to the first 4 weeks, it was much harder to read images at an early stage than to read pathologic findings in only 3 persons among total 8 patients. In a re-photographed image after 4 weeks, pathologic findings could be clearly confirmed [12]. The above studies confirmed that the accuracy and reproducibility of reading modes for qualitative panoramic images could not be perfect, and as just one wrong diagnosis may lead to a fatal prognosis, supplementary measures shall be taken. In this respect, if osteomyelitis could be diagnosed at an early stage based on a probability of 88.1% through the method used in this study, such a method could be used in the situation of dental clinicians without the need for a radiologist. Furthermore, digitalized analysis can show the ossification state indirectly by the photographic density. In this way, the method could also be used for post-operative regular checkups of osteomyelitis patients.

### Analysis of osteomyelitis subclassification

As clarified by the results of this study, suppurative type of osteomyelitis could generate a conditional inference tree using differences in the avg. value and min value of photographic density and the patient group could be classified from the normal control group based on a probability of 96.8%. Sclerosing type osteomyelitis could similarly generate a conditional inference tree using differences in avg., min, and max values of photographic density, and the patient group could be classified from normal control group based on a probability of 94.1%. As differences in photographic density of the focus site of each patient normal site are biased to one side (positive or negative side), when performing relative classification, it is considered that the error may not be significant.

When comparing BRONJ and bacterial osteomyelitis based on photographic density changes of similar aspects in panoramic radiograph through *t* test by comparison of B1 and C1 or B2 and C2, there was no significant difference in photographic density. This result means that it is not possible to classify BRONJ and bacterial osteomyelitis by quantitative analysis of panoramic radiograph. There have been many attempts to segment radiologic characteristics of BRONJ in panoramic radiograph and CT, but a clear standard could not be defined, in line with the results of this study [24–26].

A previous study mentioned that a clear standard and detailed classification are required for periosteal reaction, bone thickness and density, and hard/soft tissue changes in patients with osteomyelitis, and it is considered desirable to provide this standard through required quantitative and qualitative analysis [26].

### Differences from existing quantitative analyses

Quantitative analysis using panoramic images has been previously applied although such attempts were limited. One approach is to assume bone density using mandibular cortical width degree or index; in this method, bone density was estimated by converting a value measuring mandible margin length from mental foramen to gonial angle into the proper formula [27]. Klemetti generated a classification system of bone density at the back side of the mental foramen based on this quantitative analysis as a base in an attempt to achieve a significant outcome through quantitative analysis [28].

There was an effort to standardize imaging errors that depend on the posture of the patient at the time of radiographing. As a method of standardizing the panoramic radiograph itself at the time of photographing, there is a report of attaching a nickel stepwedge to the film cassette [29]. A recent study analyzed non-standardized panoramic radiographs using specific software. Using Digora®, bone density was analyzed by dividing it into photographic density of 254 stages in order to determine prognosis after extracting odontogenic keratocysts. Quantitative analysis of panoramic radiographs revealed that maxillary bone density 6 months after the operation showed a significant difference compared with maxillary bone density 12 months after the operation, confirming changes in bone density [30].

The quantitative analysis method for photographic density of panoramic radiograph used in the present study has the limitations that separate pre-treatment or post-treatment steps were performed for standardization and measurement was made using additional software for research. It is encouraging that more precise research results could be obtained through the above process, but it adds the disadvantage that directly utilizing data under general treatment situations is almost impossible when considering both time and resources. The significance of this study is that it shows the feasibility of measuring photographic density using only a basic function in PACS and the resulting data can help in diagnosing osteomyelitis.

### Limitations

The biggest limitation of this study is to overcome the low reproducibility of panoramic radiography itself. Photographic density, image size, or location may vary depending on photographing posture and other detailed settings, meaning that the reliability of data being obtained through quantitative analysis is questionable. For this objective, it is impossible to perform standardization for all the patients through pre-treatment. However, as mentioned previously, panoramic radiography is being widely utilized as an assistant diagnostic tool in the field for observing prognosis and concerns regarding this limitation are essentially raising doubt about using panoramic radiography itself. And also, unlike CT, which uses the same blackening leveling method in all programs, it could not be proved that our suggested program will be applicated in other digital panorama software program, because the other program will use the blackening degree relative value calculation methods only for the program itself.

The use of this method may become difficult in cases where a focus site exists in both sides, but in the present study, a focus existed in the one side of left/right in 89.5% (*n* = 85) of cases, compared with 7.4% (*n* = 7) with a focus in both left and right; therefore, this should not be a problem. In addition, designating an accurate location and size for the opposite side by bilateral symmetry after determining the location and size of the focus may require considerably skilled techniques. In this study, for consistency, the focus size was compared by placing it on the same line, but at the time of actual utilization, it could be modified so that the total focus would be included. Whether this approach would be fully functional in real applications should be considered. Excluding osteomyelitis, there are diverse diseases affecting the radiographic density and some of these diseases do not require treatment or others, on the other hand, require aggressive treatment. This is one of the biggest limitations of this clinical study designs.

It is hard to directly discriminate a very big periapical lesion, a cyst with unclear boundary or other positive tumor, and osteomyelitis only by photographic density information from a radiograph. Because the detailed entity of osteomyelitis is essential for its final treatment process, other diagnostic tools such as dental CT scanning are also essential for its discriminations. If diagnosis of osteomyelitis is performed based on the principle of considering a radiograph together with clinical diagnosis, medical history, and visual inspection at the time of diagnosis, this should not present a major program. In cases of a focus in which a change in photographic density is observed in panoramic radiograph, it would be possible to make a quantitative diagnostic standard by performing the same process.

## Conclusion

We have proposed a quantitative easy method using panoramic radiography for the early diagnosis of osteomyelitis. In particular, for clinical use, we suggest a method of quantitative analysis using the PACS program only, without the need for complicated and expensive software.

**Fig. 4:**
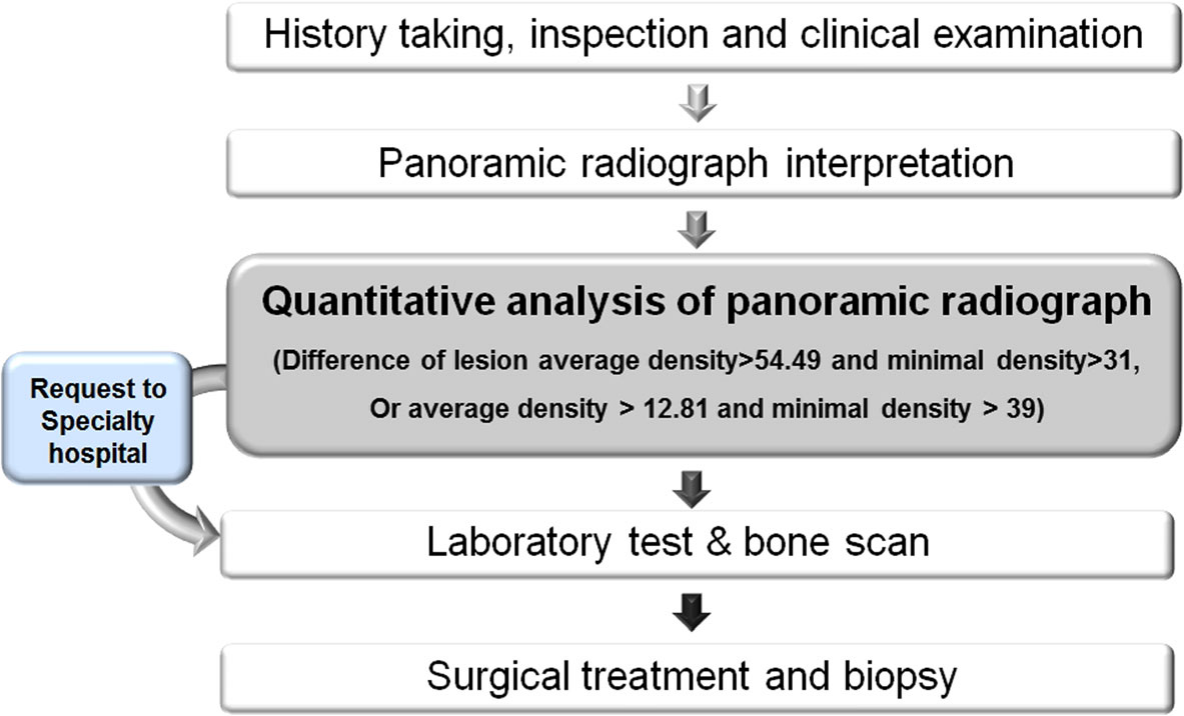
Diagnosis model using quantitative analysis of panoramic radiograph in jaw osteomyelitis suspicious patient

According to a conditional inference tree prepared in this study, in the case of a new patient showing clinical symptoms suspicious of osteomyelitis, a difference in average value greater than 54.49 and difference in minimum value less than 31 when measuring photographic density of the site is considered suspicious of osteomyelitis. On acquired panoramic radiograph, a difference in average value between 12.81 and 54.49 and difference in minimum value greater than 39 can also be suspicious of osteomyelitis (Fig. 4). Dental clinicians may refer such patients to a general hospital or confirm osteomyelitis by laboratory testing and tissue biopsy. This method is considered to be a useful aid at the time of reading images by specialists in dental clinics.

## Additional file

Additional file 1: Appendix Tables. (DOC 574 kb)

## Abbreviations

BRONJ: Bisphosphonate-related osteonecrosis of the jaw
CT: Computed tomography
DICOM: Digital Imaging and Communications in Medicine
EMR: Electronic medical record
ORN: Osteoradionecrosis
PACS: Picture archiving and communication system
ROI: Region of interest
SD: Standard deviation
