# Supplementary material for: Early diagnosis of jaw osteomyelitis by easy digitalized panoramic analysis
Source: Maxillofac Plast Reconstr Surg. 2019 Feb 1;41(1):6. doi: 10.1186/s40902-019-0188-2 (PMC6358629; doi:10.1186/s40902-019-0188-2)
Supplement: Supplementary file 1 — Appendix Tables. (DOC 574 kb) [file 40902_2019_188_MOESM1_ESM.doc]

**Appendix Tables**

Table S1. Clinicopathologic data of osteoradionecrosis patients

| **Number** | **Sex** | **Age** | **Focus** | **Clinical exam** | **Radiograph interpretation** | **Pathologic exam / Clinical diagnosis** | **Subtype** | **Treatment** | **MIN**  **(focus)** | **MAX**  **(focus)** | **AVG**  **(focus)** | **MIN**  **(WNL)** | **MAX**  **(WNL)** | **AVG**  **(WNL)** | **MIN**  **(DIF)** | **MAX**  **(DIF)** | **AVG**  **(DIF)** |
| --- | --- | --- | --- | --- | --- | --- | --- | --- | --- | --- | --- | --- | --- | --- | --- | --- | --- |
| 693373 | F | 46 | Lt&Rt mand. | ESR(H) | osteomyelitis | Osteoradionecrosis | sclerosing | partial maxillectomy | 1553 | 1972 | 1794.71 | 1646 | 2079 | 1903.48 | -93 | -107 | -108.77 |
| 703131 | M | 75 | Rt mand. | WNL | osteomyelitis | Osteoradionecrosis | sclerosing | saucerization | 1573 | 2116 | 1852.77 | 1671 | 2057 | 1938.17 | -98 | 59 | -85.4 |
| 749728 | F | 56 | Lt mand. | ESR(H), Seg. Neutrophil(H) | osteomyelitis | Osteoradionecrosis | sclerosing | saucerization | 1603 | 2102 | 1852.37 | 1675 | 2181 | 1946.99 | -72 | -79 | -94.62 |
| 858433 | M | 83 | Rt mand. | WNL | osteomyelitis | Osteoradionecrosis | sclerosing | saucerization | 1513 | 1984 | 1808.88 | 1722 | 2065 | 1913.48 | -209 | -81 | -104.6 |

Table S2. Clinicopathologic data of BRONJ patients

| **Number** | **Sex** | **Age** | **Focus** | **Clinical exam** | **Radiograph interpretation** | **Pathologic exam / Clinical diagnosis** | **Subtype** | **Treatment** | **MIN**  **(focus)** | **MAX**  **(focus)** | **AVG**  **(focus)** | **MIN**  **(WNL)** | **MAX**  **(WNL)** | **AVG**  **(WNL)** | **MIN**  **(DIF)** | **MAX**  **(DIF)** | **AVG**  **(DIF)** |
| --- | --- | --- | --- | --- | --- | --- | --- | --- | --- | --- | --- | --- | --- | --- | --- | --- | --- |
| 778971 | F | 73 | Lt mand. | WNL | osteomyelitis | BRONJ | suppurative | saucerization | 1480 | 2091 | 1896.11 | 1649 | 2020 | 1863.72 | -169 | 71 | 32.39 |
| 668160 | M | 68 | Lt mand. | ESR(H), Seg. Neutrophil(H) | osteomyelitis | BRONJ | suppurative | partial mandibulectomy | 1573 | 2076 | 1832.29 | 1468 | 2029 | 1699.15 | 105 | 47 | 133.14 |
| 744201 | F | 78 | Rt mand. | ESR(H) | osteomyelitis | BRONJ | suppurative | I&D | 1539 | 1992 | 1802.43 | 1508 | 1998 | 1711.27 | 31 | -6 | 91.16 |
| 731633 | F | 85 | Lt mand. | WNL | osteomyelitis | BRONJ | suppurative | saucerization | 1764 | 2085 | 1889.84 | 1615 | 2110 | 1809.25 | 149 | -25 | 80.59 |
| 764169 | F | 69 | Rt mand. | ESR(H) | osteomyelitis | BRONJ | suppurative | saucerization | 1854 | 2175 | 2042.27 | 1671 | 2102 | 1933.21 | 183 | 73 | 109.06 |
| 801895 | F | 72 | Rt max. | ESR(H), Seg. Neutrophil(L) | osteomyelitis | BRONJ | suppurative | saucerization | 1939 | 2372 | 2177.43 | 1882 | 2319 | 2118.33 | 57 | 53 | 59.1 |
| 852040 | F | 82 | Lt mand. | ESR(H), Monocyte(H) | osteomyelitis | BRONJ | suppurative | saucerization | 1809 | 2110 | 1980.4 | 1578 | 1975 | 1811.75 | 231 | 135 | 168.65 |
| 858916 | F | 83 | Rt mand. | ESR(H) | osteomyelitis | BRONJ | suppurative | saucerization | 1789 | 2119 | 1985.95 | 1781 | 2091 | 1954.43 | 8 | 28 | 31.52 |
| 624651 | F | 81 | Ant max. | WNL | osteomyelitis | BRONJ | suppurative | saucerization | 1694 | 2245 | 2019.39 | 1736 | 2251 | 2046.65 | -42 | -6 | -27.26 |
| 849528 | F | 82 | Rt mand. | ESR(H) | osteomyelitis | BRONJ | suppurative | saucerization | 1634 | 2074 | 1885.69 | 1412 | 1905 | 1659.8 | 222 | 169 | 225.89 |
| 786674 | F | 91 | Rt mand. | ESR(H) | osteomyelitis | BRONJ | suppurative | saucerization | 1770 | 2141 | 1946.75 | 1539 | 1992 | 1768.66 | 231 | 149 | 178.09 |
| 666784 | F | 60 | Ant mand. | ESR(H), MPV(L) | osteomyelitis | BRONJ | suppurative | saucerization | 1851 | 2282 | 2098.71 | 1792 | 2228 | 1990.94 | 59 | 54 | 107.77 |
| 715899 | F | 72 | Lt max. | ESR(H), Seg. Neutrophil(L) | osteomyelitis | BRONJ | suppurative | saucerization | 1609 | 2200 | 1975.42 | 1573 | 2026 | 1819.49 | 36 | 174 | 155.93 |
| 622596 | F | 72 | Lt&Rt mand. | WNL | osteomyelitis | BRONJ | suppurative | saucerization | 1632 | 2119 | 1904.59 | 1406 | 1941 | 1673.48 | 226 | 178 | 231.11 |
| 730285 | F | 70 | Lt mand. | ESR(H) | osteomyelitis | BRONJ | suppurative | medicine | 1634 | 2091 | 1887.19 | 1477 | 1978 | 1797.1 | 157 | 113 | 90.09 |
| 570815 | F | 52 | Rt mand. | ESR(H) | osteomyelitis | BRONJ | suppurative | saucerization | 1725 | 2051 | 1918.26 | 1601 | 2045 | 1827.81 | 124 | 6 | 90.45 |
| 700843 | F | 75 | Rt mand. | ESR(H), Seg. Neutrophil(H) | osteomyelitis | BRONJ | suppurative | saucerization | 1677 | 2034 | 1882.06 | 1573 | 1975 | 1769.75 | 104 | 59 | 112.31 |
| 771291 | F | 74 | Lt mand. | ESR(H), Seg. Neutrophil(H) | osteomyelitis | BRONJ | suppurative | saucerization | 1657 | 2057 | 1905.74 | 1471 | 1826 | 1653.17 | 186 | 231 | 252.57 |
| 782999 | F | 65 | Rt mand. | WNL | osteomyelitis | BRONJ | suppurative | medicine | 1809 | 2175 | 2002.34 | 1587 | 1939 | 1763.16 | 222 | 236 | 239.18 |
| 789300 | F | 82 | Ant mand. | ESR(H) | osteomyelitis, BRONJ | BRONJ | suppurative | sequestrectomy | 1764 | 2147 | 1973.44 | 1651 | 2026 | 1849.38 | 113 | 121 | 124.06 |
| 791175 | F | 79 | Lt max. | ESR(H) | osteomyelitis | BRONJ | suppurative | saucerization | 1632 | 2110 | 1937.68 | 1570 | 2034 | 1821.78 | 62 | 76 | 115.9 |
| 857044 | F | 68 | Rt max. | ESR(H), Seg. Neutrophil(L) | osteomyelitis | BRONJ | suppurative | saucerization | 1941 | 2352 | 2184.82 | 1733 | 2251 | 2002.18 | 208 | 101 | 182.64 |
| 794098 | F | 74 | Rt max. | ESR(H), Seg. Neutrophil(H) | osteomyelitis, malignancy | BRONJ | sclerosing | untreated. | 1511 | 2062 | 1771.1 | 1601 | 2265 | 1960.84 | -90 | -203 | -189.74 |
| 748283 | F | 61 | Rt mand. | WNL | localized osteomyelitis | BRONJ | sclerosing | saucerization | 1446 | 1820 | 1581.01 | 1744 | 2099 | 1915.54 | -298 | -279 | -334.53 |
| 756826 | F | 82 | Lt mand. | WNL | osteomyelitis | BRONJ | sclerosing | mass resection | 1553 | 1964 | 1779.51 | 1730 | 2057 | 1913.17 | -177 | -93 | -133.66 |
| 754924 | F | 78 | Rt mand. | ESR(H) | osteomyelitis | BRONJ | sclerosing | saucerization | 1482 | 1958 | 1755.99 | 1634 | 1964 | 1820.36 | -152 | -6 | -64.37 |
| 796627 | F | 70 | Rt mand. | ESR(H), Seg. Neutrophil(H) | osteomyelitis | BRONJ | sclerosing | marginal mandibulectomy | 1370 | 1840 | 1580.67 | 1527 | 2034 | 1767.44 | -157 | -194 | -186.77 |
| 771877 | F | 74 | Rt mand. | ESR(H) | osteomyelitis | BRONJ | sclerosing | saucerization | 1530 | 1902 | 1718.41 | 1744 | 2062 | 1922.01 | -214 | -160 | -203.6 |
| 761512 | F | 55 | Lt mand. | ESR(H) | osteomyelitis | BRONJ | sclerosing | saucerization | 1533 | 1964 | 1773.53 | 1848 | 2192 | 2045.58 | -315 | -228 | -272.05 |
| 689909 | F | 60 | Rt mand. | WNL | osteomyelitis, BRONJ | BRONJ | sclerosing | sequestrectomy | 1432 | 1865 | 1612.33 | 1649 | 2031 | 1889.48 | -217 | -166 | -277.15 |
| 803174 | M | 82 | Rt mand. | WNL | sclerosing osteitis | BRONJ | sclerosing | sequestrectomy | 1223 | 1716 | 1488.97 | 1460 | 1905 | 1680.08 | -237 | -189 | -191.11 |

Table S3. Clinicopathologic data of Bacterial osteomyelitis patients

| **Number** | **Sex** | **Age** | **Focus** | **Clinical exam** | **Radiograph interpretation** | **Pathologic exam / Clinical diagnosis** | **Subtype** | **Treatment** | **MIN**  **(focus)** | **MAX**  **(focus)** | **AVG**  **(focus)** | **MIN**  **(WNL)** | **MAX**  **(WNL)** | **AVG**  **(WNL)** | **MIN**  **(DIF)** | **MAX**  **(DIF)** | **AVG**  **(DIF)** |
| --- | --- | --- | --- | --- | --- | --- | --- | --- | --- | --- | --- | --- | --- | --- | --- | --- | --- |
| 607054 | F | 69 | Lt mand. | WNL | osteomyelitis | osteomyelitis | suppurative | medicine | 1908 | 2251 | 2118.31 | 1874 | 2228 | 2034.51 | 34 | 23 | 83.8 |
| 619954 | F | 80 | Lt max. | ESR(H) | osteomyelitis | osteomyelitis | suppurative | I&D | 1820 | 2274 | 2053.17 | 1677 | 2198 | 1973.82 | 143 | 76 | 79.35 |
| 705686 | M | 71 | Lt mand. | ESR(H), Seg. Neutrophil(H) | osteomyelitis | osteomyelitis | suppurative | saucerization | 1772 | 2124 | 1983.36 | 1499 | 1922 | 1737.21 | 273 | 202 | 246.15 |
| 731265 | M | 67 | Rt mand. | ESR(H) | osteomyelitis | osteomyelitis | suppurative | saucerization | 1820 | 2110 | 1987.42 | 1471 | 1840 | 1663.73 | 349 | 270 | 323.69 |
| 756169 | F | 70 | Lt max. | ESR(H) | osteomyelitis | osteomyelitis | suppurative | saucerization | 1846 | 2189 | 2026.4 | 1764 | 2175 | 2007.86 | 82 | 14 | 18.54 |
| 764446 | M | 40 | Rt mand. | ESR(H) | osteomyelitis | osteomyelitis | suppurative | saucerization | 1770 | 2158 | 2003.37 | 1713 | 2167 | 1933.95 | 57 | -9 | 69.42 |
| 785809 | F | 75 | Lt mand. | WNL | osteomyelitis | osteomyelitis | suppurative | sequestrectomy | 1603 | 2119 | 1929.1 | 1429 | 2076 | 1801.1 | 174 | 43 | 128 |
| 709453 | M | 78 | Lt mand. | ESR(H) | osteomyelitis | osteomyelitis | suppurative | sequestrectomy | 1418 | 1820 | 1614.38 | 1460 | 1834 | 1647.79 | -42 | -14 | -33.41 |
| 767260 | F | 83 | Lt&Rt mand. | ESR(H) | osteomyelitis | osteomyelitis | suppurative | untreated | 1725 | 2082 | 1933.41 | 1634 | 2065 | 1878.56 | 91 | 17 | 54.85 |
| 602179 | M | 55 | Lt mand. | MPV(H), Seg. Neutrophil(H) | osteomyelitis | osteomyelitis | suppurative | saucerization | 1696 | 2065 | 1887.69 | 1606 | 1874 | 1743.73 | 90 | 191 | 143.96 |
| 263165 | F | 55 | Rt mand. | ESR(H), Seg. Neutrophil(H) | osteomyelitis | osteomyelitis | suppurative | saucerization | 1834 | 2099 | 1971.01 | 1665 | 1978 | 1826.28 | 169 | 121 | 144.73 |
| 612123 | M | 56 | Rt mand. | ESR(H) | osteomyelitis | osteomyelitis | suppurative | saucerization | 1595 | 2212 | 1936.99 | 1756 | 2243 | 1959.19 | -161 | -31 | -22.2 |
| 639287 | M | 64 | Lt max. | ESR(H) | osteomyelitis | osteomyelitis | suppurative | partial maxillectomy | 1840 | 2324 | 2164.56 | 1663 | 2372 | 2042.84 | 177 | -48 | 121.72 |
| 67244 | F | 73 | Lt mand. | ESR(H), Seg. Neutrophil(H) | osteomyelitis | osteomyelitis | suppurative | saucerization | 1809 | 2172 | 1999.79 | 1595 | 2009 | 1805.36 | 214 | 163 | 194.43 |
| 724643 | M | 46 | Rt mand. | ESR(H), Seg. Neutrophil(H) | osteomyelitis | osteomyelitis | suppurative | saucerization | 1556 | 2003 | 1808.9 | 1415 | 1840 | 1639.52 | 141 | 163 | 169.38 |
| 767276 | M | 74 | Lt mand. | ESR(H) | osteomyelitis | osteomyelitis | suppurative | partial mandibulectomy | 1815 | 2181 | 2035.11 | 1801 | 2121 | 1980.84 | 14 | 60 | 54.27 |
| 787902 | F | 63 | Rt mand. | WNL | osteomyelitis | osteomyelitis | suppurative | saucerization | 1764 | 2144 | 1997.62 | 1519 | 2062 | 1796.23 | 245 | 82 | 201.39 |
| 791385 | M | 38 | Rt mand. | - | osteomyelitis | osteomyelitis | suppurative | Untreated. | 1685 | 2130 | 1933.05 | 1634 | 2110 | 1818.69 | 51 | 20 | 114.36 |
| 808244 | F | 76 | Rt mand. | ESR(H) | osteomyelitis, BRONJ | osteomyelitis | sclerosing | saucerization | 1280 | 1924 | 1593.86 | 1440 | 2054 | 1757.47 | -160 | -130 | -163.61 |
| 663815 | M | 11 | Rt mand. | - | periapical rarefying osteitis | osteomyelitis | sclerosing | endodontic treatment | 1688 | 2161 | 1941.12 | 1817 | 2257 | 2065.91 | -129 | -96 | -124.79 |
| 711938 | M | 37 | Rt mand. | WNL | osteomyelitis | osteomyelitis | sclerosing | saucerization | 1764 | 2183 | 1983.69 | 1812 | 2228 | 2033.55 | -48 | -45 | -49.86 |
| 710148 | M | 66 | Rt max. | ESR(H) | osteomyelitis | osteomyelitis | sclerosing | medicine | 1392 | 1882 | 1624.36 | 1460 | 1950 | 1731.44 | -68 | -68 | -107.08 |
| 724491 | F | 37 | Rt mand. | WNL | osteomyelitis | osteomyelitis | sclerosing | saucerization | 1418 | 1896 | 1676.6 | 1567 | 1958 | 1760.98 | -149 | -62 | -84.38 |
| 662009 | F | 55 | Lt max. | ESR(H) | osteomyelitis | osteomyelitis | sclerosing | sequestrectomy | 1437 | 2102 | 1813.89 | 1539 | 2217 | 2003.98 | -102 | -115 | -190.09 |
| 719918 | F | 59 | Lt&Rt max&mand. | ESR(H) | periapical rarefying osteitis | osteomyelitis | sclerosing | medicine | 1370 | 1837 | 1616.7 | 1761 | 2076 | 1932.99 | -391 | -239 | -316.29 |
| 732316 | M | 73 | Lt mand. | WNL | periapical rarefying osteitis | osteomyelitis | sclerosing | Untreated. | 1494 | 2034 | 1714.93 | 1544 | 1975 | 1773.36 | -50 | 59 | -58.43 |
| 748293 | F | 46 | Rt mand. | ESR(H), Seg. Neutrophil(L) | osteomyelitis | osteomyelitis | sclerosing | I&D | 1725 | 2062 | 1913.24 | 1877 | 2234 | 2081.31 | -152 | -172 | -168.07 |
| 763111 | M | 31 | Lt mand. | WNL | osteomyelitis | osteomyelitis | sclerosing | saucerization | 1547 | 2076 | 1752.77 | 1725 | 2079 | 1932.02 | -178 | -3 | -179.25 |
| 310137 | F | 72 | Lt mand. | WNL | osteomyelitis | osteomyelitis | sclerosing | untreated. | 1268 | 1950 | 1591.62 | 1440 | 2003 | 1664.47 | -172 | -53 | -72.85 |
| 694623 | F | 58 | Rt mand. | ESR(H) | sclerosing osteomyelitis | osteomyelitis | sclerosing | Medicine | 1646 | 1975 | 1807.21 | 1688 | 2102 | 1887.45 | -42 | -127 | -80.24 |
| 777410 | F | 65 | Lt&Rt mand. | ESR(H) | sclerosing osteomyelitis | osteomyelitis | sclerosing | saucerization | 1646 | 2065 | 1817.18 | 1877 | 2195 | 2053.85 | -231 | -130 | -236.67 |
| 721744 | F | 68 | Rt mand. | ESR(H) | osteomyelitis | osteomyelitis | sclerosing | mass resection | 1488 | 1832 | 1682.19 | 1539 | 1992 | 1801.5 | -51 | -160 | -119.31 |
| 791596 | F | 67 | Lt mand. | ESR(H), Seg. Neutrophil(L) | osteomyelitis | osteomyelitis | sclerosing | hemimandibulectomy | 1418 | 1975 | 1739.77 | 1789 | 2110 | 1952.28 | -371 | -135 | -212.51 |
| 800281 | F | 27 | Rt mand. | Seg. Neutrophil(L) | sclerosing osteitis | osteomyelitis | sclerosing | Saucerization | 1634 | 1975 | 1803.82 | 1750 | 2029 | 1903.44 | -116 | -54 | -99.62 |
| 806579 | M | 76 | Lt mand. | ESR(H), Seg. Neutrophil(L) | osteomyelitis | osteomyelitis | sclerosing | Saucerization | 1587 | 1967 | 1774.2 | 1868 | 2212 | 2078.94 | -281 | -245 | -304.74 |
| 276618 | M | 79 | Rt mand. | ESR(H), MPV(L) | osteomyelitis | osteomyelitis | sclerosing | partial mandibulectomy | 1437 | 1832 | 1598.62 | 1674 | 2012 | 1826.86 | -237 | -180 | -228.24 |
| 645836 | F | 39 | Rt mand. | WNL | osteomyelitis | osteomyelitis | sclerosing | medicine | 1741 | 2169 | 1967.1 | 1801 | 2167 | 2026.86 | -60 | 2 | -59.76 |
| 666554 | M | 62 | Rt max. | ESR(H) | periapical rarefying osteitis | osteomyelitis | sclerosing | Saucerization | 1361 | 2271 | 1780.37 | 1592 | 2251 | 1926.32 | -231 | 20 | -145.95 |
| 723283 | M | 73 | Lt mand. | ESR(H), Seg. Neutrophil(H) | osteomyelitis | osteomyelitis | sclerosing | saucerization | 1587 | 2026 | 1799.39 | 1767 | 2093 | 1953.56 | -180 | -67 | -154.17 |
| 773049 | M | 59 | Rt mand. | ESR(H) | osteomyelitis | osteomyelitis | sclerosing | saucerization | 1477 | 1868 | 1646.73 | 1587 | 1910 | 1740.57 | -110 | -42 | -93.84 |
| 787940 | M | 71 | Lt mand. | WNL | osteomyelitis | osteomyelitis | sclerosing | saucerization | 1494 | 1950 | 1706.82 | 1547 | 2026 | 1820.08 | -53 | -76 | -113.26 |
| 811104 | F | 78 | Rt mand. | ESR(H) | sclerosing osteitis | osteomyelitis | sclerosing | saucerization | 1246 | 2051 | 1570.51 | 1975 | 2237 | 2110.6 | -729 | -186 | -540.09 |
| 860223 | M | 70 | Rt mand. | WNL | osteomyelitis | osteomyelitis | sclerosing | saucerization | 1502 | 1975 | 1708.55 | 1837 | 2195 | 2063.25 | -335 | -220 | -354.7 |
| 705034 | F | 69 | Rt mand. | ESR(H) | osteomyelitis | osteomyelitis | sclerosing | cyst enucleation | 1322 | 1778 | 1509.59 | 1578 | 1891 | 1742.38 | -256 | -113 | -232.79 |
| 674308 | M | 48 | Lt mand. | Seg. Neutrophil(L) | osteomyelitis | osteomyelitis | sclerosing | untreated. | 1440 | 1834 | 1620.08 | 1502 | 1936 | 1716.86 | -62 | -102 | -96.78 |
| 649094 | M | 65 | Rt mand. | ESR(H), MPV(H), Seg. Neutrophil(L) | periapical rarefying osteitis | osteomyelitis | sclerosing | untreated. | 1761 | 2212 | 1994.63 | 1832 | 2243 | 2071.86 | -71 | -31 | -77.23 |
| 658742 | M | 33 | Lt mand. | WNL | periapical rarefying osteitis | osteomyelitis | sclerosing | extraction | 1584 | 2164 | 1868.78 | 1573 | 1995 | 1820.09 | 11 | 169 | 48.69 |
| 324612 | F | 47 | Lt mand. | - | periapical rarefying osteitis | osteomyelitis | sclerosing | untreated. | 1530 | 1950 | 1760.78 | 1466 | 2045 | 1739.86 | 64 | -95 | 20.92 |
| 689332 | F | 63 | Lt&Rt mand. | ESR(H) | periapical rarefying osteitis | osteomyelitis | sclerosing | extraction | 1770 | 2133 | 1982.82 | 1677 | 2040 | 1889.43 | 93 | 93 | 93.39 |
| 627145 | M | 51 | Lt&Rt mand. | ESR(H), Seg. Neutrophil(H) | osteomyelitis | osteomyelitis | sclerosing | medicine | 1418 | 1913 | 1639.51 | 1556 | 2015 | 1827.55 | -138 | -102 | -188.04 |
| 167324 | F | 34 | Lt mand. | - | osteomyelitis | osteomyelitis | sclerosing | untreated. | 1663 | 2000 | 1822.31 | 1764 | 2082 | 1943.35 | -101 | -82 | -121.04 |
| 796801 | M | 63 | Lt mand. | ESR(H), Seg. Neutrophil(L) | osteomyelitis | osteomyelitis | sclerosing | untreated. | 1412 | 1820 | 1629.77 | 1443 | 1978 | 1748.75 | -31 | -158 | -118.98 |
| 809916 | F | 39 | Rt mand. | - | sclerosing osteitis,osteomyelitis | osteomyelitis | sclerosing | extraction | 1688 | 2127 | 1920.91 | 2003 | 2251 | 2155.04 | -315 | -124 | -234.13 |
| 790618 | M | 60 | Rt mand. | Seg. Neutrophil(L) | sclerosing osteitis | osteomyelitis | sclerosing | untreated. | 1626 | 2020 | 1841.67 | 1764 | 2150 | 1998.19 | -138 | -130 | -156.52 |
| 794062 | M | 57 | Rt mand. | WNL | sclerosing osteitis | osteomyelitis | sclerosing | untreated. | 1423 | 1848 | 1633.69 | 1609 | 2057 | 1886.32 | -186 | -209 | -252.63 |
| 781773 | F | 29 | Lt mand. | WNL | osteomyelitis | osteomyelitis | sclerosing | saucerization | 1634 | 1961 | 1796.78 | 1764 | 2062 | 1917.23 | -130 | -101 | -120.45 |
| 532982 | M | 68 | Rt mand. | - | osteomyelitis | osteomyelitis | sclerosing | cyst enucleation | 1499 | 1939 | 1711.31 | 1615 | 2051 | 1876.03 | -116 | -112 | -164.72 |
| 808423 | F | 80 | Rt mand. | ESR(H) | osteomyelitis, BRONJ | osteomyelitis | sclerosing | saucerization | 1764 | 2082 | 1947.37 | 1457 | 2119 | 1860.85 | 307 | -37 | 86.52 |
| 663189 | F | 27 | Rt mand. | WNL | osteomyelitis | osteomyelitis | sclerosing | saucerization | 1423 | 1908 | 1636.31 | 1573 | 2099 | 1894.66 | -150 | -191 | -258.35 |
| 808245 | M | 60 | Lt mand. | Seg. Neutrophil(L) | osteomyelitis | osteomyelitis | sclerosing | cyst enucleation | 1820 | 2099 | 1959.02 | 1657 | 2015 | 1864.01 | 163 | 84 | 95.01 |

Table S4. Data of control group (WNL)

| **Number** | **Sex** | **Age** | **MIN(WNL1)** | **MAX(WNL1)** | **AVG(WNL1)** | **MIN(WNL2)** | **MAX(WNL2)** | **AVG(WNL2)** | **MIN(DIF)** | **MAX(DIF)** | **AVG(DIF)** |
| --- | --- | --- | --- | --- | --- | --- | --- | --- | --- | --- | --- |
| 620090 | F | 38 | 1710 | 2234 | 2081.82 | 1792 | 2245 | 2069.97 | -82 | -11 | 11.85 |
| 617934 | M | 29 | 1840 | 2203 | 2043.01 | 1789 | 2181 | 2020.19 | 51 | 22 | 22.82 |
| 615528 | M | 59 | 1823 | 2240 | 2080.76 | 1764 | 2228 | 2060.56 | 59 | 12 | 20.2 |
| 615029 | F | 19 | 1922 | 2231 | 2088.84 | 1888 | 2231 | 2076.7 | 34 | 0 | 12.14 |
| 596791 | M | 34 | 1958 | 2248 | 2166.46 | 1953 | 2209 | 2116.82 | 5 | 39 | 49.64 |
| 611514 | F | 23 | 1888 | 2189 | 2072.71 | 1868 | 2189 | 2077.25 | 20 | 0 | -4.54 |
| 601067 | F | 64 | 2029 | 2276 | 2189.66 | 2012 | 2274 | 2180.79 | 17 | 2 | 8.87 |
| 608329 | F | 23 | 1961 | 2214 | 2102.65 | 1941 | 2299 | 2163.47 | 20 | -85 | -60.82 |
| 267110 | M | 49 | 1829 | 2186 | 2037.16 | 1843 | 2192 | 2026.28 | -14 | -6 | 10.9 |
| 638855 | F | 68 | 1725 | 2085 | 1933.25 | 1764 | 2133 | 1972.41 | -39 | -48 | -39.16 |
| 605312 | F | 55 | 1832 | 2228 | 2065.94 | 1772 | 2223 | 2038.93 | 60 | 5 | 27.01 |
| 625925 | F | 26 | 1440 | 2378 | 2080.72 | 1401 | 2352 | 2055.75 | 39 | 26 | 24.97 |
| 601292 | M | 68 | 1725 | 2147 | 1953.13 | 1725 | 2178 | 1993.57 | 0 | -31 | -40.44 |
| 567056 | M | 59 | 1832 | 2279 | 2059.72 | 1806 | 2228 | 2028.4 | 26 | 51 | 31.32 |
| 632994 | M | 50 | 1874 | 2248 | 2090.13 | 1865 | 2231 | 2091.87 | 9 | 17 | -1.74 |
| 582373 | F | 75 | 1640 | 1978 | 1824.01 | 1609 | 1975 | 1832.61 | 31 | 3 | -8.6 |
| 582780 | M | 77 | 1651 | 2133 | 1885.55 | 1629 | 2138 | 1880.4 | 22 | -5 | 5.15 |
| 661968 | F | 53 | 1882 | 2259 | 2121.18 | 1801 | 2265 | 2110.68 | 81 | -6 | 10.5 |
| 667722 | M | 21 | 1891 | 2192 | 2062.22 | 1910 | 2195 | 2068.61 | -19 | -3 | -6.39 |
| 643105 | M | 50 | 1573 | 2017 | 1825.19 | 1525 | 2091 | 1836.37 | 48 | -74 | -11.18 |
| 595011 | M | 29 | 1820 | 2203 | 2027.96 | 1803 | 2200 | 2037.63 | 17 | 3 | -9.67 |
| 666553 | M | 35 | 1665 | 2057 | 1874.59 | 1620 | 2076 | 1862.65 | 45 | -19 | 11.94 |
| 666541 | M | 38 | 1803 | 2127 | 1966.23 | 1795 | 2102 | 1965.92 | 8 | 25 | 0.31 |
| 660893 | F | 49 | 1710 | 2290 | 2025.34 | 1702 | 2302 | 2003.58 | 8 | -12 | 21.76 |
| 650447 | M | 55 | 1680 | 2324 | 2104.51 | 1609 | 2310 | 2105.47 | 71 | 14 | -0.96 |
| 665206 | M | 43 | 1781 | 2192 | 2028.47 | 1809 | 2155 | 2010.36 | -28 | 37 | 18.11 |
| 665182 | M | 30 | 1834 | 2228 | 2083.15 | 1860 | 2228 | 2071.68 | -26 | 0 | 11.47 |
| 436420 | F | 56 | 1772 | 2167 | 1975.79 | 1750 | 2121 | 1976.05 | 22 | 46 | -0.26 |
| 615762 | F | 43 | 1792 | 2217 | 2023.51 | 1789 | 2240 | 2038.97 | 3 | -23 | -15.46 |
| 650467 | F | 54 | 1770 | 2302 | 2124.08 | 1702 | 2341 | 2136.89 | 68 | -39 | -12.81 |
| 656637 | F | 70 | 1722 | 2068 | 1897.95 | 1702 | 2062 | 1902.05 | 20 | 6 | -4.1 |
| 635676 | M | 60 | 1789 | 2172 | 2024.78 | 1786 | 2209 | 2029.36 | 3 | -37 | -4.58 |
| 696150 | F | 58 | 1446 | 1913 | 1689.71 | 1446 | 1891 | 1693.53 | 0 | 22 | -3.82 |
| 678743 | F | 72 | 1696 | 2040 | 1895.2 | 1702 | 2034 | 1893.01 | -6 | 6 | 2.19 |
| 694042 | F | 30 | 1654 | 2158 | 2039.96 | 1851 | 2192 | 2022.48 | -197 | -34 | 17.48 |
| 679418 | F | 32 | 1480 | 2082 | 1870 | 1457 | 2228 | 1895.35 | 23 | -146 | -25.35 |
| 626671 | F | 56 | 1634 | 1908 | 1790.07 | 1632 | 1964 | 1792.95 | 2 | -56 | -2.88 |
| 229756 | F | 73 | 1654 | 1930 | 1802.53 | 1634 | 1939 | 1791.15 | 20 | -9 | 11.38 |
| 700488 | M | 45 | 1851 | 2265 | 2071.26 | 1784 | 2307 | 2079.75 | 67 | -42 | -8.49 |
| 729542 | F | 45 | 1508 | 1967 | 1766.05 | 1513 | 1984 | 1775.16 | -5 | -17 | -9.11 |
| 711348 | F | 49 | 1677 | 2274 | 2050.16 | 1651 | 2279 | 2060.9 | 26 | -5 | -10.74 |
| 654430 | M | 47 | 1477 | 2060 | 1733.54 | 1511 | 2062 | 1763.87 | -34 | -2 | -30.33 |
| 704920 | F | 38 | 1578 | 1953 | 1801.61 | 1550 | 1953 | 1808.38 | 28 | 0 | -6.77 |
| 541407 | F | 64 | 1615 | 1958 | 1801.49 | 1587 | 1964 | 1776.44 | 28 | -6 | 25.05 |
| 702583 | M | 79 | 1936 | 2178 | 2065.37 | 1924 | 2200 | 2088.88 | 12 | -22 | -23.51 |
| 544874 | F | 86 | 1750 | 2147 | 1984.43 | 1722 | 2133 | 1973.67 | 28 | 14 | 10.76 |
| 184029 | F | 77 | 1634 | 2209 | 2040.12 | 1615 | 2262 | 2022.53 | 19 | -53 | 17.59 |
| 725867 | F | 40 | 1634 | 2012 | 1870.53 | 1668 | 2040 | 1898.95 | -34 | -28 | -28.42 |
| 743087 | M | 83 | 1471 | 1860 | 1693.59 | 1519 | 1896 | 1713.74 | -48 | -36 | -20.15 |
| 742434 | F | 21 | 1832 | 2164 | 2010.15 | 1801 | 2189 | 2019.63 | 31 | -25 | -9.48 |
| 734370 | M | 36 | 1632 | 2144 | 1966.93 | 1629 | 2214 | 1948.25 | 3 | -70 | 18.68 |
| 498189 | M | 31 | 1615 | 2062 | 1842.32 | 1615 | 1950 | 1804.77 | 0 | 112 | 37.55 |
| 608780 | M | 80 | 1888 | 2186 | 2055.8 | 1877 | 2141 | 2020 | 11 | 45 | 35.8 |
| 473758 | F | 73 | 1632 | 1916 | 1793.5 | 1626 | 1939 | 1797.19 | 6 | -23 | -3.69 |
| 791905 | M | 19 | 1888 | 2167 | 2026.85 | 1854 | 2158 | 2036.89 | 34 | 9 | -10.04 |
| 744853 | M | 30 | 1770 | 2200 | 2015.1 | 1795 | 2220 | 2069.59 | -25 | -20 | -54.49 |
| 747097 | M | 53 | 1761 | 2133 | 1990.42 | 1795 | 2113 | 1983.54 | -34 | 20 | 6.88 |
| 757955 | M | 68 | 1767 | 2065 | 1927.24 | 1750 | 2110 | 1960.6 | 17 | -45 | -33.36 |
| 703826 | M | 43 | 1888 | 2167 | 2046.43 | 1910 | 2169 | 2063.01 | -22 | -2 | -16.58 |
| 764027 | F | 57 | 1626 | 1910 | 1761.54 | 1646 | 1936 | 1790.9 | -20 | -26 | -29.36 |
| 726286 | F | 56 | 1716 | 2234 | 2021.52 | 1733 | 2274 | 2004.78 | -17 | -40 | 16.74 |
| 787854 | F | 58 | 1584 | 2127 | 1909.86 | 1595 | 2147 | 1975.36 | -11 | -20 | -65.5 |
| 334142 | F | 37 | 1702 | 2068 | 1910.98 | 1702 | 2068 | 1913.02 | 0 | 0 | -2.04 |
| 774777 | M | 28 | 1680 | 2000 | 1848.71 | 1674 | 2012 | 1855.64 | 6 | -12 | -6.93 |
| 779285 | F | 31 | 1705 | 1961 | 1840.62 | 1713 | 2009 | 1877.94 | -8 | -48 | -37.32 |
| 360421 | M | 66 | 1792 | 2121 | 1991.82 | 1744 | 2141 | 1977.12 | 48 | -20 | 14.7 |
| 780336 | M | 31 | 1733 | 2015 | 1886.34 | 1699 | 1995 | 1858.43 | 34 | 20 | 27.91 |
| 713521 | F | 73 | 1815 | 2158 | 2009.5 | 1823 | 2169 | 2055.21 | -8 | -11 | -45.71 |
| 771861 | M | 25 | 1930 | 2172 | 2070.77 | 1927 | 2150 | 2049.03 | 3 | 22 | 21.74 |
| 779895 | F | 56 | 1587 | 1964 | 1838.21 | 1595 | 1964 | 1805.86 | -8 | 0 | 32.35 |
| 706375 | M | 50 | 1595 | 1992 | 1850.86 | 1587 | 1992 | 1839.24 | 8 | 0 | 11.62 |
| 745025 | M | 64 | 1984 | 2220 | 2111.51 | 1930 | 2240 | 2123.3 | 54 | -20 | -11.79 |
| 801933 | M | 50 | 1443 | 1888 | 1683.3 | 1423 | 1865 | 1677.44 | 20 | 23 | 5.86 |
| 782424 | F | 72 | 1480 | 2065 | 1776.76 | 1492 | 2048 | 1749.56 | -12 | 17 | 27.2 |
| 555106 | M | 42 | 1471 | 1896 | 1678.15 | 1415 | 1882 | 1645.34 | 56 | 14 | 32.81 |
| 704776 | F | 55 | 1494 | 1865 | 1681.65 | 1519 | 1891 | 1692.4 | -25 | -26 | -10.75 |
| 802601 | M | 37 | 1308 | 1823 | 1557.79 | 1280 | 1826 | 1555.14 | 28 | -3 | 2.65 |
| 808233 | F | 31 | 1418 | 1905 | 1598.03 | 1406 | 1920 | 1597.81 | 12 | -15 | 0.22 |
| 801936 | M | 56 | 1761 | 2091 | 1953.89 | 1747 | 2082 | 1944.59 | 14 | 9 | 9.3 |
| 803262 | F | 62 | 1764 | 2093 | 1940.46 | 1761 | 2110 | 1956.02 | 3 | -17 | -15.56 |
| 746003 | F | 75 | 1713 | 2026 | 1879.31 | 1736 | 2031 | 1882.99 | -23 | -5 | -3.68 |
| 810784 | M | 48 | 1404 | 1860 | 1658.61 | 1423 | 1860 | 1624.16 | -19 | 0 | 34.45 |
| 808256 | M | 50 | 1511 | 1865 | 1693.33 | 1519 | 1868 | 1691.94 | -8 | -3 | 1.39 |
| 804423 | F | 80 | 1865 | 2189 | 2056.62 | 1840 | 2183 | 2040.35 | 25 | 6 | 16.27 |
| 775209 | F | 52 | 1722 | 1992 | 1867.41 | 1736 | 2023 | 1917.66 | -14 | -31 | -50.25 |
| 803599 | M | 23 | 1798 | 2062 | 1954.79 | 1781 | 2045 | 1937.9 | 17 | 17 | 16.89 |
| 808721 | M | 21 | 1877 | 2198 | 2049 | 1888 | 2167 | 2027.24 | -11 | 31 | 21.76 |
| 646298 | M | 90 | 1730 | 2003 | 1880.06 | 1713 | 2045 | 1908.67 | 17 | -42 | -28.61 |
| 860869 | M | 49 | 1595 | 2003 | 1817.07 | 1527 | 2093 | 1815.86 | 68 | -90 | 1.21 |
| 860844 | F | 52 | 1603 | 1975 | 1823.32 | 1603 | 2031 | 1836.15 | 0 | -56 | -12.83 |
| 740307 | M | 67 | 1429 | 2110 | 1817.26 | 1412 | 2142 | 1829.44 | 17 | -32 | -12.18 |
| 866295 | F | 27 | 1649 | 1950 | 1819.89 | 1646 | 1975 | 1843.14 | 3 | -25 | -23.25 |
| 711348 | F | 53 | 1423 | 1984 | 1725.49 | 1412 | 2014 | 1745.34 | 11 | -30 | -19.85 |
| 865850 | M | 20 | 1778 | 2085 | 1953.36 | 1792 | 2105 | 1949.92 | -14 | -20 | 3.44 |
| 865358 | F | 33 | 1716 | 2034 | 1897.73 | 1730 | 2043 | 1906.14 | -14 | -9 | -8.41 |
| 843952 | F | 23 | 1786 | 2093 | 1980.39 | 1764 | 2082 | 1960.97 | 22 | 11 | 19.42 |
| 368044 | M | 27 | 1243 | 2228 | 1831.37 | 1257 | 2259 | 1802.87 | -14 | -31 | 28.5 |
| 713492 | F | 60 | 1801 | 2085 | 1961.48 | 1764 | 2099 | 1986.71 | 37 | -14 | -25.23 |
| 850877 | F | 67 | 1685 | 2003 | 1864.49 | 1663 | 2009 | 1843.53 | 22 | -6 | 20.96 |
| 675860 | M | 56 | 1542 | 2116 | 1783.01 | 1618 | 2029 | 1862.75 | -76 | 87 | -79.74 |
| 716467 | M | 12 | 1553 | 2074 | 1854.67 | 1550 | 2085 | 1891.02 | 3 | -11 | -36.35 |
| 286504 | F | 20 | 1978 | 2310 | 2208.66 | 1927 | 2355 | 2239.87 | 51 | -45 | -31.21 |
| 773087 | M | 42 | 1595 | 2012 | 1814.97 | 1710 | 2096 | 1936.98 | -115 | -84 | -122.01 |
| 759839 | M | 50 | 1651 | 2093 | 1870.72 | 1634 | 2110 | 1952.78 | 17 | -17 | -82.06 |
| 652972 | M | 36 | 1764 | 2228 | 1968.12 | 1736 | 2240 | 2057.2 | 28 | -12 | -89.08 |
| 676360 | F | 21 | 1620 | 2065 | 1892.27 | 1626 | 1986 | 1812.13 | -6 | 79 | 80.14 |
| 865377 | M | 80 | 1553 | 1927 | 1740.77 | 1482 | 1955 | 1737.49 | 71 | -28 | 3.28 |
| 859554 | M | 41 | 1702 | 2091 | 1940.71 | 1730 | 2065 | 1938.36 | -28 | 26 | 2.35 |
| 655031 | F | 24 | 1511 | 1998 | 1815.43 | 1550 | 2034 | 1834.78 | -39 | -36 | -19.35 |
| 706396 | M | 41 | 1359 | 1854 | 1572.31 | 1381 | 1806 | 1604.04 | -22 | 48 | -31.73 |
| 658749 | F | 62 | 1806 | 2251 | 2064.22 | 1817 | 2265 | 2061.87 | -11 | -14 | 2.35 |
| 689329 | F | 52 | 1375 | 1750 | 1572.66 | 1406 | 1733 | 1585.05 | -31 | 17 | -12.39 |
| 803239 | F | 47 | 1815 | 2178 | 2013.8 | 1837 | 2155 | 2027.66 | -22 | 23 | -13.86 |
| 672166 | M | 55 | 1556 | 2119 | 1817.8 | 1587 | 2169 | 1886.51 | -31 | -50 | -68.71 |
| 613292 | F | 76 | 1674 | 2082 | 1897.74 | 1632 | 2072 | 1875.4 | 42 | 10 | 22.34 |
| 599369 | M | 78 | 1553 | 1840 | 1696.09 | 1561 | 1862 | 1714.31 | -8 | -22 | -18.22 |
| 404644 | M | 44 | 1784 | 2209 | 2002.75 | 1803 | 2178 | 1978.59 | -19 | 31 | 24.16 |

Table S5. Location of osteomyelitis lesion

| **Classification**  **of lesion**  **Location**  **of lesion** | **Osteoradionecrosis** | **BRONJ** | **Bacterial osteomyelitis** | **Total (Osteomyelitis)** |
| --- | --- | --- | --- | --- |
| Rt mand. | 2 | 14 | 28 | 44 |
| Lt mand. | 1 | 8 | 21 | 30 |
| Rt max. | 0 | 3 | 2 | 5 |
| Lt max. | 0 | 2 | 4 | 6 |
| Ant mand. | 0 | 2 | 0 | 2 |
| Ant max. | 0 | 1 | 0 | 1 |
| Lt&Rt mand. | 1 | 1 | 4 | 6 |
| Lt&Rt max&mand. | 0 | 0 | 1 | 1 |
| **Total** | 4 | 31 | 60 | 95 |

Table S6. Significance verification of comparison between osteomyelitis patient and control group(WNL)

|  | | **Predicted** | | | | | |
| --- | --- | --- | --- | --- | --- | --- | --- |
|  |  | WNL | OM | WNL | Suppurative  OM | WNL | Sclerosing  OM |
| **Actual** | WNL | 16 | 0 | 23 | 0 | 23 | 2 |
|  | Osteomyelitis | 5 | 21 | 3 | 5 | 0 | 9 |
| **Accuracy** | | 88.1% | | 96.8% | | 94.1% | |

Table S7. Statistical significance verification of average photographic density difference (Avg) between Suppurative type osteomyelitis and Sclerosing type osteomyelitis

| **Independent Samples Test** | | | | | | | | | | |
| --- | --- | --- | --- | --- | --- | --- | --- | --- | --- | --- |
|  | | **Levene's Test for Equality of Variances** | | **t-test for Equality of Means** | | | | | | |
|  |  | **F** | **Sig.** | **t** | **df** | **Sig.**  **(2-tailed)** | **Mean Difference** | **Std. Error Difference** | **95% Confidence Interval of the Difference** | |
|  |  |  |  |  |  |  |  |  | **Lower** | **Upper** |
| **avg** | **Equal variances assumed** | 3.019 | 0.086 | 12.625 | 89.000 | 0.000 | 274.623 | 21.752 | 231.403 | 317.843 |
|  | **Equal variances not assumed** |  |  | 13.195 | 87.562 | 0.000 | 274.623 | 20.813 | 233.258 | 315.988 |

p < 0.01, reject null hypothesis and there is difference between two groups

Table S8. Statistical significance verification of maximum photographic density difference (Max) between Suppurative type osteomyelitis and Sclerosing type osteomyelitis

| **Independent Samples Test** | | | | | | | | | | |
| --- | --- | --- | --- | --- | --- | --- | --- | --- | --- | --- |
|  | | **Levene's Test for Equality of Variances** | | **t-test for Equality of Means** | | | | | | |
|  |  | **F** | **Sig.** | **t** | **df** | **Sig. (2-tailed)** | **Mean Difference** | **Std. Error Difference** | **95% Confidence Interval of the Difference** | |
|  |  |  |  |  |  |  |  |  | **Lower** | **Upper** |
| **avg** | **Equal variances assumed** | 0.047 | 0.829 | 10.133 | 89.000 | 0.000 | 188.088 | 18.562 | 151.205 | 224.971 |
|  | **Equal variances not assumed** |  |  | 10.280 | 87.572 | 0.000 | 188.088 | 18.297 | 151.724 | 224.452 |

p < 0.01, reject null hypothesis and there is difference between two groups

Table S9. Statistical significance verification of minimum photographic density difference (Min) between Suppurative type osteomyelitis and Sclerosing type osteomyelitis

| **Independent Samples Test** | | | | | | | | | | |
| --- | --- | --- | --- | --- | --- | --- | --- | --- | --- | --- |
|  | | **Levene's Test for Equality of Variances** | | **t-test for Equality of Means** | | | | | | |
|  |  | **F** | **Sig.** | **t** | **df** | **Sig. (2-tailed)** | **Mean Difference** | **Std. Error Difference** | **95% Confidence Interval of the Difference** | |
|  |  |  |  |  |  |  |  |  | **Lower** | **Upper** |
| **avg** | **Equal variances assumed** | 0.718 | 0.399 | 9.182 | 89.000 | 0.000 | 262.924 | 28.633 | 206.030 | 319.817 |
|  | **Equal variances not assumed** |  |  | 9.549 | 88.365 | 0.000 | 262.924 | 27.533 | 208.211 | 317.636 |

p < 0.01, reject null hypothesis and there is difference between two groups

Table S10. Statistical significance verification of average photographic density difference (Avg) among suppurative type (B1 / C1) in BRONJ and bacterial osteomyelitis

| **Independent Samples Test** | | | | | | | | | | |
| --- | --- | --- | --- | --- | --- | --- | --- | --- | --- | --- |
|  | | **Levene's Test for Equality of Variances** | | **t-test for Equality of Means** | | | | | | |
|  |  | **F** | **Sig.** | **t** | **df** | **Sig. (2-tailed)** | **Mean Difference** | **Std. Error Difference** | **95% Confidence Interval of the Difference** | |
|  |  |  |  |  |  |  |  |  | **Lower** | **Upper** |
| **avg** | **Equal variances assumed** | 0.641 | 0.428 | 0.398 | 38.000 | 0.693 | 10.315 | 25.887 | -42.090 | 62.721 |
|  | **Equal variances not assumed** |  |  | 0.389 | 32.232 | 0.699 | 10.315 | 26.485 | -43.618 | -64.249 |

p > 0.01, accept null hypothesis and there is no difference between two groups

Table S11. Statistical significance verification of maximum photographic density difference (Max) among suppurative type (B1 / C1) in BRONJ and bacterial osteomyelitis

| **Independent Samples Test** | | | | | | | | | | |
| --- | --- | --- | --- | --- | --- | --- | --- | --- | --- | --- |
|  | | **Levene's Test for Equality of Variances** | | **t-test for Equality of Means** | | | | | | |
|  |  | **F** | **Sig.** | **t** | **df** | **Sig. (2-tailed)** | **Mean Difference** | **Std. Error Difference** | **95% Confidence Interval of the Difference** | |
|  |  |  |  |  |  |  |  |  | **Lower** | **Upper** |
| **avg** | **Equal variances assumed** | 0.810 | 0.374 | 0.686 | 38.000 | 0.497 | 17.980 | 26.226 | -35.112 | 71.072 |
|  | **Equal variances not assumed** |  |  | 0.672 | 32.921 | 0.506 | 17.980 | 26.745 | -36.438 | 72.398 |

p > 0.01, accept null hypothesis and there is no difference between two groups

Table S12. Statistical significance verification of minimum photographic density difference (Min) among suppurative type (B1 / C1) in BRONJ and bacterial osteomyelitis

| **Independent Samples Test** | | | | | | | | | | |
| --- | --- | --- | --- | --- | --- | --- | --- | --- | --- | --- |
|  | | **Levene's Test for Equality of Variances** | | **t-test for Equality of Means** | | | | | | |
|  |  | **F** | **Sig.** | **t** | **df** | **Sig. (2-tailed)** | **Mean Difference** | **Std. Error Difference** | **95% Confidence Interval of the Difference** | |
|  |  |  |  |  |  |  |  |  | **Lower** | **Upper** |
| **avg** | **Equal variances assumed** | 0.320 | 0.575 | -0.084 | 38.000 | 0.934 | -2.949 | 35.286 | -74.382 | 68.483 |
|  | **Equal variances not assumed** |  |  | -0.082 | 33.730 | 0.935 | -2.949 | 35.842 | -75.811 | 69.912 |

p > 0.01, accept null hypothesis and there is no difference between two groups

Table S13. Statistical significance verification of average photographic density difference (Avg) among sclerosing type (B2 / C2) in BRONJ and bacterial osteomyelitis

| **Independent Samples Test** | | | | | | | | | | |
| --- | --- | --- | --- | --- | --- | --- | --- | --- | --- | --- |
|  | | **Levene's Test for Equality of Variances** | | **t-test for Equality of Means** | | | | | | |
|  |  | **F** | **Sig.** | **t** | **df** | **Sig. (2-tailed)** | **Mean Difference** | **Std. Error Difference** | **95% Confidence Interval of the Difference** | |
|  |  |  |  |  |  |  |  |  | **Lower** | **Upper** |
| **avg** | **Equal variances assumed** | 1.032 | 0.315 | -1.515 | 49.000 | 0.136 | -64.579 | 42.627 | -150.240 | 21.082 |
|  | **Equal variances not assumed** |  |  | -1.968 | 16.923 | 0.066 | -64.579 | 32.811 | -133.828 | 4.670 |

p > 0.01, accept null hypothesis and there is no difference between two groups

Table S14. Statistical significance verification of maximum photographic density difference (Max) among sclerosing type (B2 / C2) in BRONJ and bacterial osteomyelitis

| **Independent Samples Test** | | | | | | | | | | |
| --- | --- | --- | --- | --- | --- | --- | --- | --- | --- | --- |
|  | | **Levene's Test for Equality of Variances** | | **t-test for Equality of Means** | | | | | | |
|  |  | **F** | **Sig.** | **t** | **df** | **Sig. (2-tailed)** | **Mean Difference** | **Std. Error Difference** | **95% Confidence Interval of the Difference** | |
|  |  |  |  |  |  |  |  |  | **Lower** | **Upper** |
| **avg** | **Equal variances assumed** | 0.337 | 0.564 | -2.446 | 49.000 | 0.018 | -79.024 | 32.314 | -143.961 | -14.087 |
|  | **Equal variances not assumed** |  |  | -2.652 | 12.798 | 0.020 | -79.024 | 29.803 | -143.513 | -14.534 |

p > 0.01, accept null hypothesis and there is no difference between two groups

Table S15. Statistical significance verification of minimum photographic density difference (Min) among sclerosing type (B2 / C2) in BRONJ and bacterial osteomyelitis

| **Independent Samples Test** | | | | | | | | | | |
| --- | --- | --- | --- | --- | --- | --- | --- | --- | --- | --- |
|  | | **Levene's Test for Equality of Variances** | | **t-test for Equality of Means** | | | | | | |
|  |  | **F** | **Sig.** | **t** | **df** | **Sig. (2-tailed)** | **Mean Difference** | **Std. Error Difference** | **95% Confidence Interval of the Difference** | |
|  |  |  |  |  |  |  |  |  | **Lower** | **Upper** |
| **avg** | **Equal variances assumed** | 1.667 | 0.203 | -1.274 | 49.000 | 0.209 | -71.048 | 55.777 | -183.135 | 41.040 |
|  | **Equal variances not assumed** |  |  | -2.048 | 28.671 | 0.050 | -71.048 | 34.686 | -142.025 | -0.070 |

p > 0.01, accept null hypothesis and there is no difference between two groups
